# Dissecting the two mechanisms of scramble competition among the Virunga mountain gorillas

**DOI:** 10.1007/s00265-021-03016-1

**Published:** 2021-04-22

**Authors:** Andrew M. Robbins, Cyril C. Grueter, Didier Abavandimwe, Tara S. Stoinski, Martha M. Robbins

**Affiliations:** 1 Max Planck Institute for Evolutionary Anthropology, Leipzig, Germany; 2 The Dian Fossey Gorilla Fund International, Atlanta, GA USA; 3 School of Human Sciences, The University of Western Australia, Perth, Australia; 4 Centre for Evolutionary Biology, School of Biological Sciences, The University of Western Australia, Perth, Australia; 5 International Centre of Biodiversity and Primate Conservation, Dali University, Dali, 671003 Yunnan China; 6 Zoo Atlanta, Atlanta, GA USA

**Keywords:** Activity budget, Travel, Contest competition, Group spread, Patch depletion, Patch residence time

## Abstract

**Abstract:**

Two mechanisms have been proposed to explain why scramble competition can increase the travel requirements of individuals within larger groups. Firstly, individuals in larger groups may be more likely to encounter food sites where other group members have already eaten, leading to greater asynchronous “individual” travel to find fresh sites. Secondly, when food sites are aggregated into patches, larger groups may need to visit more patches to obtain the same amount of food per capita, leading to greater synchronous “group” travel between patches. If the first mechanism can be mitigated by increasing group spread, then we expect the second mechanism to be more sensitive to group size. Here, we examine the individual travel and group travel of the Virunga mountain gorillas, along with potential implications for the two mechanisms of scramble competition. Asynchronous individual travel accounted for 67% of the total travel time, and the remainder arose from group travel. Group spread increased significantly for larger groups, but not enough to prevent an increase in individual travel. Contrary to expectations, group travel decreased with size among most groups, and we found only limited evidence of patch depletion that would cause the second mechanism of scramble competition. Collectively, our results illustrate how the influence of group size can differ for individual travel versus group travel, just as it differs among species for overall travel. Studies that distinguish between the two mechanisms of scramble competition may enhance our understanding of ecological constraints upon group size, including potential differences between frugivores and folivores.

**Significance statement:**

Feeding competition provides insight into how group size can influence the foraging patterns of social animals, but two key mechanisms are not typically compared. Firstly, larger groups may visit more patches to access the same amount of food per capita (group travel). Secondly, their individuals may also need to move past more spots where another member has already eaten (individual travel). Contrary to expectations, we found that group travel decreased with size for most groups of mountain gorillas, which may reflect extra travel by smaller groups to avoid larger groups. Individual travel increased with size in most groups, even though gorillas in larger groups compensated by spreading out over a broader area. The two mechanisms revealed patterns that were not apparent in our previous study of overall travel. Our approach may help to explain potential differences between folivores and frugivores.

## Introduction

Feeding competition is one of the main factors that determines how group size and habitat quality can influence the foraging patterns, reproductive success, and social structure of animals (van Schaik 1989; Clutton-Brock and Janson 2012). Feeding competition can manifest in two ways: contest competition and scramble competition (Nicholson 1954; Janson and van Schaik 1988; Sterck et al. 1997; Isbell and Young 2002). Contest competition occurs when an individual obtains a greater share of the food by excluding other individuals. Scramble competition occurs when an individual reduces the amount of food available to other individuals, simply by consuming it (van Schaik 1989; Koenig 2002; Snaith and Chapman 2007). Both types of competition can occur within groups and between groups (Janson and van Schaik 1988).

Within-group scramble competition (WGS) is predicted to reduce the foraging efficiency of all of group members, leading to greater travel for larger groups (van Schaik 1989; Koenig 2002; Snaith and Chapman 2007). Two mechanisms have been proposed to explain the increased travel (Waser 1977; van Schaik and van Hooff 1983; van Schaik et al. 1983; Gillespie and Chapman 2001). Firstly, individuals in larger groups may become more likely to encounter food sites where other group members have already eaten, leading to increased individual travel to find a fresh site. Secondly, when food sites are aggregated into patches, larger groups may need to visit more food patches to obtain the same amount of food per capita, leading to increased group travel between patches. To reflect those two mechanisms, the term “food site” will refer to a spot where an individual can feed without traveling, and “patch” will generally refer to a collection of food sites where a group can feed without traveling together (Altmann 1974; Chapman 1988; Chancellor and Isbell 2009b). Numerous studies have examined the relationship between group size and overall travel, but the relative importance of each mechanism is not typically quantified (Janson and Goldsmith 1995; Majolo et al. 2008).

Distinctions between the two mechanisms of WGS may be insightful because they can have differing impacts on the relationship between group size and travel. In particular, the first mechanism may be mitigated by increasing the spacing between individuals and/or the overall group spread (Gillespie and Chapman 2001; Hirsch 2007; Saj and Sicotte 2007; Snaith and Chapman 2008; Chancellor and Isbell 2009b). If so, then individuals may have greater spacing when feeding than resting, and groups with more individuals may have greater group spread (Gillespie and Chapman 2001; Heesen et al. 2015). Conversely, greater spacing within a group may increase the risks of predation and/or infanticide by males outside the group (Watts 1991; Smith et al. 2005; Di Blanco and Hirsch 2006). Thus, the optimal group spread may involve a trade-off between feeding competition versus external threats (Cowlishaw 1999). Several measures have been used to represent group spread, but the concept does not have a specific definition (Watts 1991; Koenig et al. 1998; Gillespie and Chapman 2001; Saj and Sicotte 2007; Snaith and Chapman 2008; Chancellor and Isbell 2009a; Heesen et al. 2015).

The second mechanism of WGS is expected to occur when groups deplete the food within a patch before leaving it. The marginal value theorem predicts that foraging efficiency will drop as the food becomes depleted, and that individuals will leave the patch when their foraging efficiency falls below the average rate that is available among all patches (Charnov 1976). If feeding competition has an equal effect upon all individuals within a group (as predicted for scramble competition), then they may leave the patch together within a short time period (Kotler et al. 1994). Even if individuals are affected unequally, or if they have different nutritional requirements, the group may still leave the patch relatively simultaneously to maintain social cohesion (Kazahari 2014). Thus, the second mechanism may lead to coordinated movements of all group members (synchronous group travel), whereas the first mechanism can allow individuals to move more independently (asynchronous individual travel). The marginal value theorem may not apply if groups leave a patch to balance their nutritional requirements, to avoid predators, or to minimize competition with other groups (Searle et al. 2005b; Harris 2006; Johnson et al. 2017). Those alternative explanations for synchronous group travel illustrate that it is not necessarily an indication of scramble competition.

Rather than rigorously testing the quantitative predictions of the marginal value theorem, most studies have merely looked for potential evidence. For example, studies have examined whether intake rates decline as a patch becomes depleted, while foraging effort increases or remains constant (Snaith and Chapman 2005). A declining intake rate has been considered evidence of patch depletion, whereas a declining foraging effort could indicate that the individuals are merely sated (Snaith and Chapman 2005). Studies of patch depletion have also examined whether group patch residence times are shorter for larger groups, smaller patches, and/or lower food density (Tombak et al. 2012; Kazahari 2014; Johnson et al. 2017). More direct tests of the marginal value theorem have considered whether the foraging efficiency is consistent across patch types when individuals leave (Grether et al. 1992; Searle et al. 2005a). One of the potential challenges for all of those methods has been to define the boundaries of a patch, and a wide variety of approaches have been attempted (Chapman 1988; Jiang and Hudson 1993; Edenius et al. 2002; Fortin et al. 2002; Searle et al. 2005a; Sayers et al. 2010; Marshall et al. 2013; Plante et al. 2014).

**Fig. 1:**
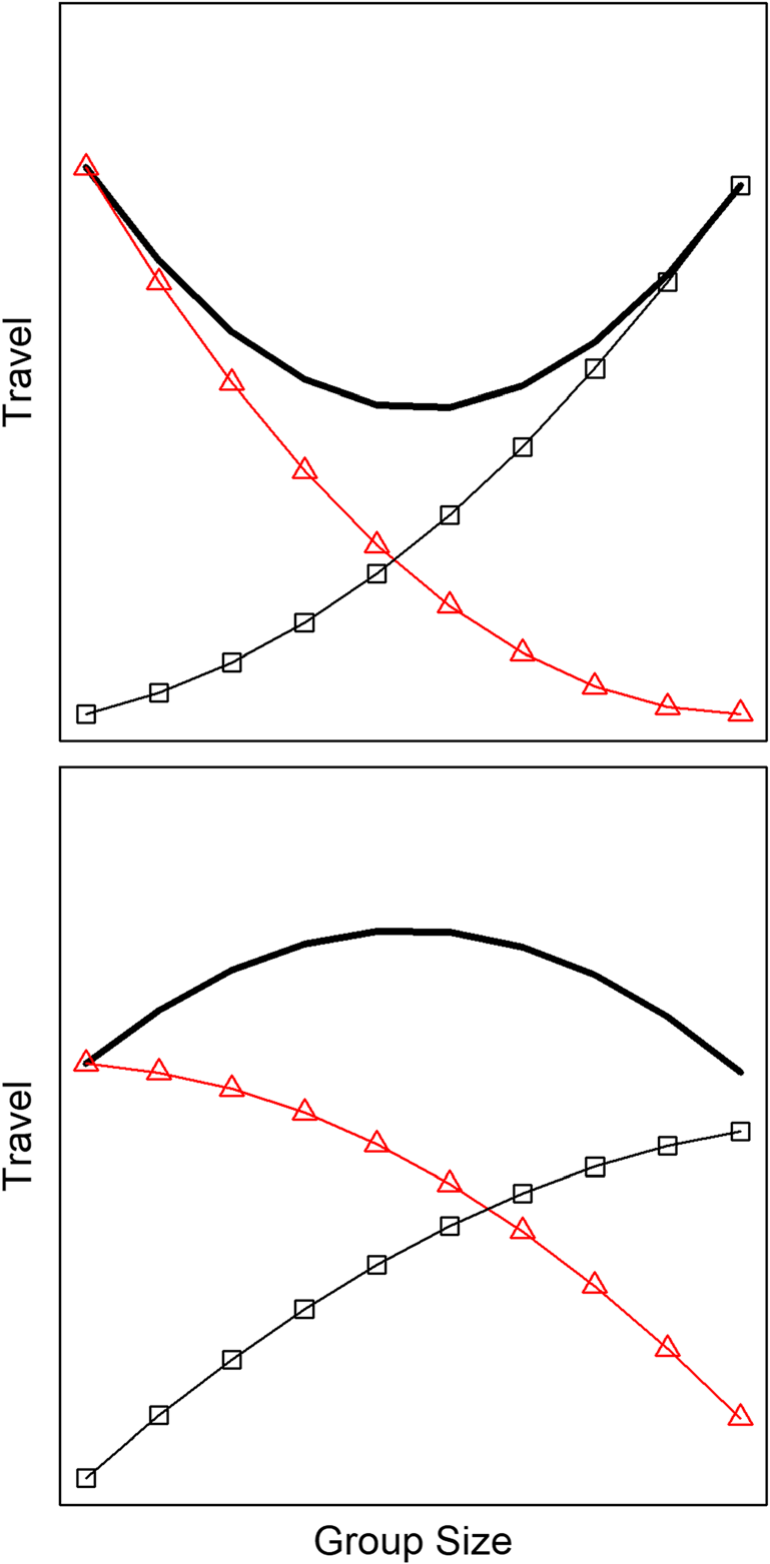
Hypothetical effects of group size on travel requirements (taken from Grueter et al. 2018). The overall travel requirements (thick line) equal the combined impact of competition within groups (circles) and between groups (triangles). The overall pattern is U-shaped if the second derivative is positive for both types of competition (1a), versus an inverted U-shape if the second derivatives are negative (1b). **a** Resembles the overall travel requirements for woolly monkeys (*Lagothrix lagothricha*) and savanna baboons (*Papio cynocephalus*), whereas **b** resembles the overall travel for mountain gorillas (Grueter et al. 2018). **a** Also resembles the results for synchronous group travel in this study, whereas **b** resembles our results for asynchronous individual travel

If increasing group spread can mitigate only the first mechanism of WGS, then the second mechanism may be more sensitive to changes in group size. If so, then differences in group size could have greater impact on synchronous group travel than asynchronous individual travel. The impact of group size upon travel may be further complicated, however, by between-group contest competition (BGC). BGC could create a negative correlation between group size and synchronous group travel, if small groups travel farther to avoid encounters with large groups, or if they are displaced from patches where such encounters occur (Wrangham 1980; Janson and Goldsmith 1995; Majolo et al. 2008; Markham et al. 2013). BGC could also create a negative correlation between group size and asynchronous individual travel, if small groups are relegated to lower quality habitats (but see Teichroeb and Sicotte 2018). A combination of WGS and BGC can lead to nonlinear patterns between group size and travel, which can have either a U-shape or an inverted-U, depending on subtle differences in the influence of each type of competition (Fig. 1). Thus, the overall impact of group size upon each type of travel can be difficult to predict.

The Virunga mountain gorillas are an interesting study population for dissecting the two mechanisms of scramble competition because they have both synchronous and asynchronous travel. The gorillas primarily feed on herbaceous vegetation which covers the ground in most areas, and often grows up to 1–2 m tall. While the group is feeding, a gorilla will occasionally leave a food site and advance a few meters to another site (asynchronous individual travel). Repeated movements can create a separate path for each gorilla, although they sometimes follow a path made by others and then branch off to their own feeding site (Grueter et al. 2016). Collectively, those individual movements produce a network of narrow, trampled trails, with food removed at some places (Watts 1998). If larger groups do not increase their spread sufficiently, then those individual paths could become more likely to overlap and/or crisscross, and individuals could face a greater probability of encountering sites where other gorillas have already eaten (Watts 1991).

In addition to the asynchronous individual travel, an entire group occasionally stops feeding and moves together (synchronous group travel). Gorillas often form a single file line during synchronous group travel, and they seem to travel faster than during asynchronous individual travel (personal observation). Groups occasionally travel through or around noticeably poor vegetation (e.g., fields of *Crassocephalum*), but more typically, it is not apparent why they are not feeding on the vegetation they traverse. If they are leaving one patch to find another one, then the vegetation between patches may have only slightly lower food quality, rather than no food at all.

Previous studies of the Virunga mountain gorillas have focused on the combined effects of both synchronous and asynchronous travel (Watts 1988, 1991; Robbins et al. 2007, 2009). Most recently, our companion study found an inverted U-shaped relationship between group size versus overall travel distances and times (Grueter et al. 2018). Travel requirements increased with group size for most groups, which was attributed to scramble competition within those groups. Surprisingly, however, travel requirements decreased slightly for the largest group, which was attributed to competition among groups (Grueter et al. 2018). Encounters between groups occur only about once a month, even when we include auditory interactions at distances up to 500 m, so any advantage for larger groups in intergroup competition does not arise by routinely displacing competitors from patches of food (Sicotte 1993; Mirville 2018; Mirville et al. 2020). Instead, smaller groups may have avoided contest competition with the largest group, which had exclusive use of 80% of its home range, compared with less than 20% for most other groups during this study (Waser 1976; Sicotte 1993; Caillaud et al. 2014). Smaller groups may be avoiding male mating competition rather than feeding competition, because intergroup encounters often involve confrontations among males (Sicotte 1993; Robbins and Sawyer 2007; Seiler et al. 2017).

The group spread of mountain gorillas is greater when feeding than resting, as expected to mitigate the first mechanism of scramble competition, but the effects of group size have not been reported (Fossey and Harcourt 1977; Grueter et al. 2016). Studies of individual spacing have mainly focused on social relationships within and among the age-sex classes (Harcourt 1979a, b; Watts 1992, 1994b; Rosenbaum et al. 2016). Female mountain gorillas compete for proximity to the dominant male, who provides protection from infanticide and predation, and is often positioned near the center of the group (Watts 1994a; Harcourt and Stewart 2007). Such proximity may also help to maintain group cohesion to reduce predation risk and/or to remain competitive in case of intergroup encounters. Watts (1991) proposed that growing groups might initially increase their spread to mitigate scramble competition, but they would eventually have to start to travel farther instead. Those predictions have not been tested, and the impact of group size on each type of travel has not been reported (Watts 1984, 1985).

Here, we examine the synchronous and asynchronous travel of the Virunga mountain gorillas, along with the two mechanisms of within-group scramble competition (Table 1). Based on those two mechanisms, we would expect that both types of travel will increase with group size. If groups increase their spread to mitigate the first mechanism of scramble competition, then we expect that group size will have less impact on asynchronous individual travel than synchronous group travel.

**Table 1:** Summary of the statistical models. In the column for predictor variables, the symbol “(2)” after group size indicates that a quadratic term was included to examine the potential combination of scramble competition and intergroup competition (which is predicted to create a quadratic relationship). Due to limited data, two models used a categorical variable for group size. Predictions for a positive (+) or negative (-) correlation are based on the first mechanism of scramble competition (s1), the second mechanism of scramble competition (s2), or intergroup competition (ig). The “results” column indicates whether the correlation was positive (+), negative (-), non-linear (“NL”), or not significant (“NS”). *p*-values are based on the “anova” function in R function to compare the full model with a null model that excluded all predictors simultaneously

Response variable	Predictor variable	Predictions		Results	*N*	R^2^	Χ^2^	df	*p*
Synchronous travel time	Group size (2)	+	s2	NL	1590	0.139	11.9	2	0.003
Asynchronous travel time	Group size (2)	+	s1	NL	1892	0.125	9.0	2	0.011
Asynchronous travel distances	Group size category	+	s1	NS	2162	0.014	0.6	1	0.457
Synchronous travel distances	Group size category	-	ig	NS	311	0.036	0.0	1	0.845
Group area	Group size	+	s1	+	1974	0.222	15.4	1	0.000
Group density	Group size	+	s1	+	1974	0.465	32.8	1	0.000
Group patch residence time	Group size (2)	-	s2	NL	1329	NA	5.3	2	0.070
Energy intake rate	Time until group travel	+	s2	+	1244	0.317	7.9	1	0.005
Intake per food site	Time until group travel	+	s2	+	1244	0.036	32.5	1	0.000
Food site residence times	Time until group travel	+	s2	+	1244	0.053	60.7	1	0.000
Asynchronous travel distances	Time until group travel	-	s2	NS	772	0.018	0.2	1	0.672
Energy intake rate	Time since group travel	-	s2	NS	1438	0.357	0.4	1	0.512
Intake per food site	Time since group travel	-	s2	+	1438	0.007	8.8	1	0.003
Food site residence times	Time since group travel	-	s2	+	1438	0.020	26.9	1	0.000
Asynchronous travel distances	Time since group travel	+	s2	-	1044	0.025	5.7	1	0.017

To examine whether large groups have greater spread than small groups, we estimated the size of the area that encompassed a group, and the density of gorillas within that area. If the group spread represents a trade-off between the first mechanism of scramble competition versus external threats, then we expect that the impact of group size will reflect a compromise between those competing factors (Table 1). We predict that large groups will occupy a greater area than small groups, but the increase will not be enough to completely avoid higher gorilla density (nor to avoid at least some increase in asynchronous individual travel).

To look for potential evidence that groups deplete patches, we hypothesized that gorillas were leaving a patch whenever they began synchronous group travel, and that they had reached a new patch when they stop traveling and resume feeding. If so, then we expect that group patch residence times will have a negative correlation with group size, and that foraging efficiency will decline as groups deplete the food within a patch. Reduced foraging efficiency could mean that gorillas have lower energy intake rates; they could spend less time (and obtain less energy) per food site; and/or they could travel farther between food sites (Table 1). Alternatively, if synchronous group travel occurs for other reasons (above) and food is more evenly distributed, then we expect foraging efficiency to remain constant for individuals within the same group.

If the two mechanisms of within-group scramble competition are complicated by competition between groups, then we predict that quadratic patterns will arise between group size versus asynchronous individual travel, synchronous group travel, and group patch residence times (Table 1). Intergroup competition could also allow the largest group to have shorter distances of synchronous travel, if it was less likely to encounter areas where other groups had already eaten.

## Methods

### Data collection

From October 2009 through December 2010, we studied nine groups of mountain gorillas that are monitored by the Karisoke Research Center of the Dian Fossey Gorilla Fund in the Volcanoes National Park of Rwanda (Table 2). The Virunga Volcano region contains a range of habitats that were classified according to their vegetation and altitude: mixed forest, bamboo forest, saddle, meadows, brush ridge, herbaceous, subalpine, and alpine (Grueter et al. 2012). Our “activity/proximity protocol” consisted of 50-min focal observations of adult females, during which we performed a point sample every 10 min to record the main activity of the group (feeding, traveling, resting, grooming, or playing), the activity of the focal female, and the number of other weaned gorillas within 5 m of proximity. For three of the groups (PAB, BWE, & NTA), we also performed a “feeding protocol” that involved focal sampling of adult females for 30-min intervals, during which we measured their foraging efficiency and the distances of synchronous group travel. As required by the Rwanda Development Board, all observations were limited to 4 h per day to minimize anthropogenic disturbance. It was not possible to record data blind because our study involved focal animals in the field.

**Table 2:** Sample sizes for each of the groups in each of the models in Figs. 2 and 3. Figure 2a and b had the same sample sizes

Group	Fig. 2	Fig. 3a	Fig. 3b	Fig. 3c
BWE	244	219	131	121
INS	95	90	71	47
ISA	337	328	183	174
KUY	184	181	188	86
NTA	211	202	165	268
PAB	309	301	302	279
TIT	160	158	183	106
UGE	313	297	277	76
URU	121	116	90	172

Data for foraging efficiency were taken from 3342 food sites in our previous study (Grueter et al. 2018). The food site residence time (FSRT) was defined as the elapsed time from when a female began eating, until she stopped eating and/or moved more than 1 m (Chancellor and Isbell 2009b; Wright et al. 2014). The energy intake for each food site equaled the number of food items that the gorilla ate, multiplied by the average energy content for each item (Rothman et al. 2007; Nakagawa 2009). The energy intake rate equaled the total energy intake divided by the FSRT. The distances of individual asynchronous travel between food sites excluded cases that included behavior other than foraging (e.g., resting), as well as cases when the group was traveling synchronously.

### Analyses of group spread and asynchronous individual travel

Our analyses of group density and group area were based on a circle with a radius of 5 m around each focal female (Table 3). For each 10-min point sample in the activity/proximity protocol, we estimated the “experienced density” of the focal female as number of weaned gorillas within that circle (including the focal female), divided by the area of the circle (78.5 m^2^). For example, if we found two weaned gorillas within 5 m of the focal female, then there would be three weaned gorillas within the 5-m circle (the focal female plus the other two gorillas). The experienced density would equal: (3 weaned gorillas) / (78.5 m^2^) = 0.038 weaned gorillas per square meter. We estimated the “total occupied group area” as the area of the circle, divided by the proportion of weaned gorillas from the group that were in the circle. For example, if the group contained 15 weaned gorillas, then the three gorillas in the previous example would represent 20% of the group. The estimated group area would equal: (78.5 m^2^) / 0.2 = 392.5 m^2^.

**Table 3:** Additional details for the analyses of total occupied group area and experienced density. Sample sizes (N) for each group. Mean and standard deviation for the total number of weaned individuals per group (“weaned”), the number of weaned individuals that were still immature (“immature”), the number of weaned individuals within 5 m of the focal female (“proximity”), and the proportion of weaned individuals that were within the 5-m radius for proximity measurements (“proportion”). The “proximity” variable does not include the focal female but the “proportion” variable does (see Methods)

Group	*N*	Weaned	Immature	Proximity	Proportion
BWE	244	7.0 ± 0.6	0.00 ± 0.00	0.93 ± 1.18	0.28 ± 0.17
INS	95	4.0 ± 0.0	0.91 ± 0.29	1.05 ± 1.01	0.51 ± 0.25
ISA	337	7.0 ± 0.0	1.20 ± 0.59	1.60 ± 0.98	0.37 ± 0.14
KUY	184	10.9 ± 0.7	2.00 ± 0.00	0.81 ± 1.08	0.17 ± 0.10
NTA	211	9.0 ± 0.0	2.89 ± 0.76	1.62 ± 1.27	0.29 ± 0.14
PAB	309	38.8 ± 0.4	21.25 ± 0.52	3.93 ± 2.71	0.13 ± 0.07
TIT	160	6.0 ± 0.6	3.77 ± 0.42	1.59 ± 1.43	0.43 ± 0.24
UGE	313	11.3 ± 1.0	3.96 ± 0.72	1.10 ± 1.46	0.19 ± 0.13
URU	121	3.2 ± 0.6	0.12 ± 0.33	0.79 ± 0.91	0.56 ± 0.27

If focal observations are representative of an entire group, then the experienced density of a focal individual will reflect the overall density of the group, and the total occupied group area will reflect the actual area of the group. The experienced density is proportional to the number of weaned gorillas within the circle, so either variable would produce the same results in our analyses. We chose to present the density variable because it seems more meaningful than the number of weaned gorillas within the circle (i.e., if other researchers used a different radius, they would probably need a conversion to make a meaningful comparison with the number of weaned gorillas within the circle). Although our variables for the experienced density and the total occupied group area provide two perspectives on the same raw data, their relationships with group size can vary separately. If the experienced density increases with group size, for example, the total occupied group area can increase, decrease, or remain constant.

To test the hypothesis that larger groups increase their group spread to mitigate the potential costs of the first mechanism of scramble competition, we ran a linear mixed model in which the response variable was the total occupied group area during the scans while the focal female was feeding. For each day of the study, the model contained a separate data point for each focal female in each habitat where she was feeding. The predictor variable was the number of weaned gorillas in the group (i.e., group size). We ran a similar model in which the response variable was the experienced density instead of the total occupied group area.

To examine the proportion of time that adult females spent on asynchronous individual travel, we ran a linear mixed model with one data point for each focal female in each habitat on each day. For each data point, the response variable equaled the number of 10-min point samples when the focal female was traveling asynchronously (i.e., while the rest of the group was not traveling). A pseudo-offset variable equaled the total number of point samples for the focal female in the habitat on the day. Thus, the model is essentially predicting the number of times that a female was observed traveling, while controlling for the total number of times that the female was observed. Our approach is similar to using the proportion of time spent traveling asynchronously as the response variable, but the pseudo-offset variable helps to avoid excessive influence from data points that are based on fewer observations (McCullagh and Nelder 2008). The predictor variables in the model were group size and size squared. The term for group size squared was included to evaluate potentially nonlinear relationships between group size and travel requirements (Fig. 1). We also controlled for daily rainfall because the gorillas typically stop traveling and feeding when it is raining (Watts 1991; Ganas and Robbins 2005; Grueter et al. 2018). The model used data from the activity/proximity protocol.

To examine the distances of asynchronous individual travel between consecutive food sites, we ran a model with one data point for each site. The response variable was the distance that the female traveled to reach the site (meters). Food site data was collected for only three groups, which did not include the two smallest groups, which limited our ability to look for nonlinear effects of group size. Instead, we used a categorical variable which merely tested whether the largest group (PAB) was significantly different from two intermediate sized groups (NTA and BWE). The model used data from the feeding protocol.

### Analyses of synchronous group travel and patch utilization

To examine the proportion of time that gorillas spent on synchronous group travel, we ran a linear mixed model with one data point for each habitat that each group used on each day. The response variable equaled the duration of time that the group spent on synchronous travel, with a pseudo-offset variable for the total duration of observations. The predictor variables were the size of the group and size squared. Again, the term for group size squared was included to evaluate potentially nonlinear relationships between group size and travel requirements (Fig. 1). The model used data from the activity/proximity protocol which indicated when travel was the main activity of the group.

Based on our hypothesis of gorilla food patches, we defined the “group patch residence time” as the duration of feeding between when a group stops traveling synchronously (potentially entering a new patch) until the group resumes traveling synchronously again (potentially leaving a patch). To examine whether larger groups had shorter group patch residence times, we ran a mixed-effect Cox model with one data point for each time that a group stopped traveling synchronously (potentially entering a new patch). The response variable was the number of point samples that feeding was the main group activity, so resting did not contribute to the group patch residence times. A residence time began when a point sample indicated that travel was no longer the main activity of the group (i.e., when group travel ended). The residence time ended when the observations stopped, or when a point sample indicated that travel had once again become the main activity of the group. Data points were right-censored if observations ended before synchronous group travel resumed (i.e., before the group potentially left the patch). The predictor variables were the size of the group and size squared. The model used data from the activity/proximity protocol.

To examine whether groups deplete patches of food, we tested whether foraging efficiency was declining before they resumed synchronous group travel (potentially leaving a patch). We ran four models that each used one data point for each food site that was observed before the synchronous group travel (except for the final food site). In order to remain with the rest of the group, the female could have left the final site before it was depleted, so results for the final sites might not fully reflect the marginal value theorem. The response variables for the first three models were the distance traveled to reach the food site (meters), the time spent feeding at the food site (minutes), and the total energy intake at the food site (kJ). To examine the energy intake rate (kJ per minute), the fourth model used the total energy intake at the food site (kJ) as the response variable, with an offset variable for the time spent feeding at the site (minutes). In each of those four models, the predictor variable was the (log transformed) time remaining until the synchronous group travel resumed. We ran another set of four models in which the predictor variable was the (log transformed) time since a group stopped synchronous travel (potentially entering a new patch). This two-pronged approach enabled us to use data when we did not observe the entire group patch residence time. Both sets of models used data from the feeding protocol.

To evaluate the distances of synchronous group travel, we ran a model with one data point for each time that the entire journey was observed. The response variable was the distance (meters) between the food sites where the focal female was feeding immediately before and after the synchronous group travel. The predictor variable was a categorical variable which indicated whether the group was large or intermediate-sized. Data for the model was obtained through the feeding protocol.

### Statistical details

Random effect variables for the mixed models included the identity of the group and the type of habitat. From October through December, the gorillas consumed bamboo shoots in two of the habitats (mixed forest and bamboo). To account for those seasonal variations in food availability, those two habitats were subdivided into separate categories for the bamboo season versus the rest of the year (Grueter et al. 2012). When the model included separate data points for each female, we added the identity of the female as another random effect variable, which helped to control for differences among females including any influence of dominance rank (Grueter et al. 2016). The linear mixed models also included a control variable for temporal autocorrelation among the data points (Furtbauer et al. 2011).

All linear mixed models were run with a Gaussian error structure and identity link function while using the “lmer” function of the “lme4” package in R (Bates et al. 2015). We used log or exponential transformations of the response variables as needed to obtain normally distributed residuals. The predictor variables for group size were log transformed to provide a more uniform distribution of values, and then were standardized so they each had a mean value of “0” and a standard deviation of “1” (Schielzeth 2010). We used the “anova” function to establish the overall statistical significance of each model (Table 1), by comparing it with a null model that excluded all predictor variables simultaneously (Schielzeth and Forstmeier 2009). We calculated the R^2^ values for the full models by using the function “r.squaredGLMM” in the R package “MuMIn” (Table 1). We obtained *p*-values for each predictor variable by using the “drop1” function (Tables 4, 5, 6, and 7), which performs likelihood ratio tests to compare the full model versus a set of reduced models that exclude one predictor at a time (Barr et al. 2013). Error terms are presented as ± one standard deviation (SD).

## Results

### Group spread and asynchronous individual travel

**Fig. 2:**
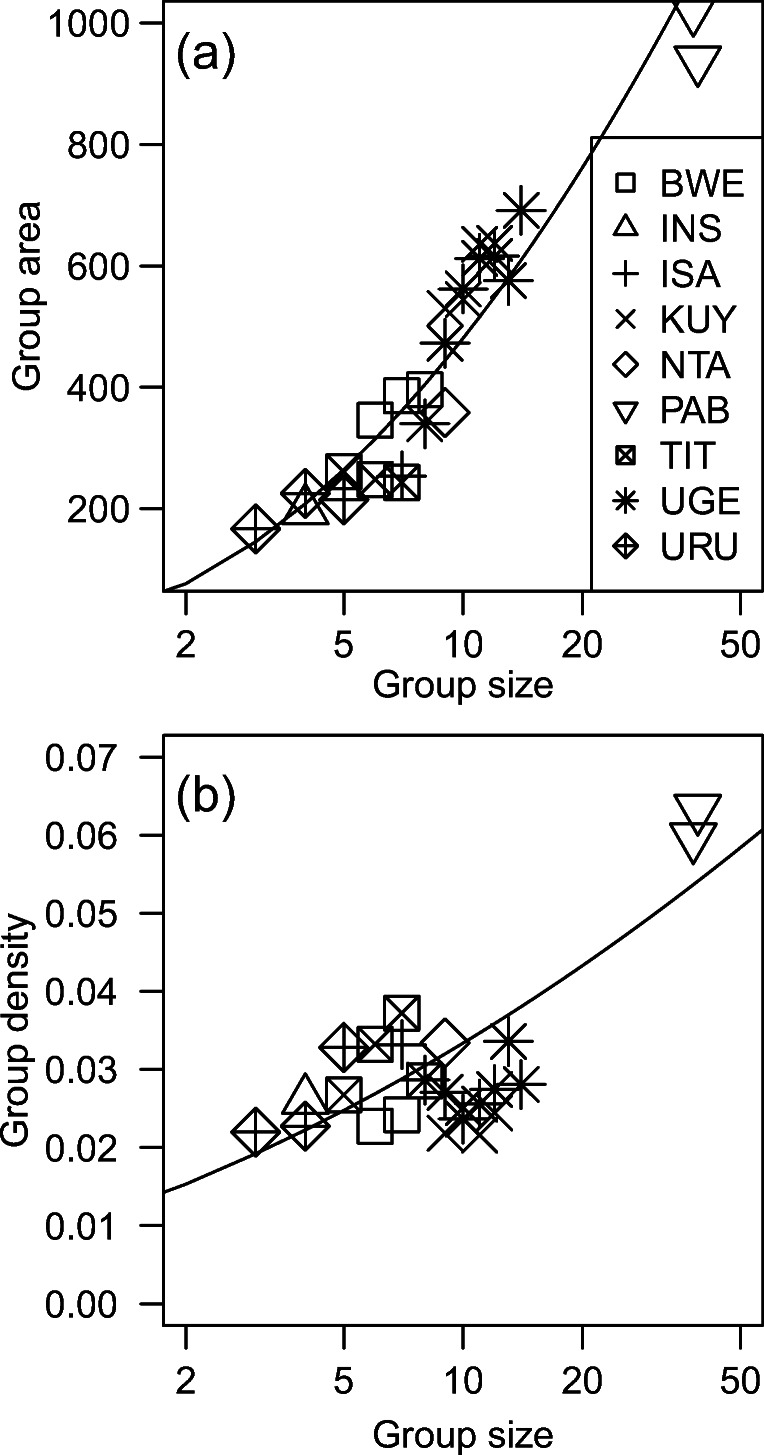
Estimates of the total occupied group area (square meters) and experienced density (weaned gorillas per square meter) versus the number of weaned gorillas in the group. The x-axes have a log-scale, and the y-axes have a linear scale. Lines are based on linear regressions. Each data point represents a different size of a different group. See Tables 2 and 3 for more details

Groups had an estimated “total occupied group area” of 472.2 ± 408.5 SD square meters while feeding, and they had an estimated “experienced density” of 0.034 ± 0.021 weaned gorillas per square meter (Table 3). The number of weaned gorillas in the group (i.e., group size) had a significant positive correlation with our estimates of total occupied group area and experienced density (Table 4, Fig. 2). Visual inspection suggested that the correlation between experienced density and group size could be heavily influenced by the largest group (Fig. 2b), but the correlation remained significant when we excluded that group from post hoc analysis (*p* = 0.016). Collectively, these results indicate that larger groups were spread over a greater area than smaller groups (as expected to mitigate the first mechanism of scramble competition), even though their gorillas were spaced more tightly.

**Table 4:** Results from statistical models for group spread and asynchronous individual travel. *p*-values are excluded for control variables

Fixed effect	Estimate	StdErr	*t*	*p*
a) Group area while feeding				
Group size	5.043	0.562	8.975	< 0.001
Autocorrelation	0.962	0.116	8.258	–
b) Gorilla density within groups while feeding				
(Intercept)	0.180	0.007	26.356	–
Group size	0.022	0.005	4.190	< 0.001
Autocorrelation	0.008	0.001	9.174	–
c) Time spent on asynchronous travel				
(Intercept)	0.765	0.099	7.723	–
Total time	0.249	0.019	12.930	–
Group size	0.414	0.131	3.169	0.003
Size squared	− 0.222	0.076	− 2.918	0.005
Rainfall	− 0.033	0.016	− 2.030	–
Autocorrelation	0.040	0.016	2.425	–
d) Travel distances between consecutive food sites				
(Intercept)	1.066	0.032	33.493	–
Size category	0.034	0.048	0.714	0.457
Autocorrelation	0.105	0.019	5.500	–

**Fig. 3:**
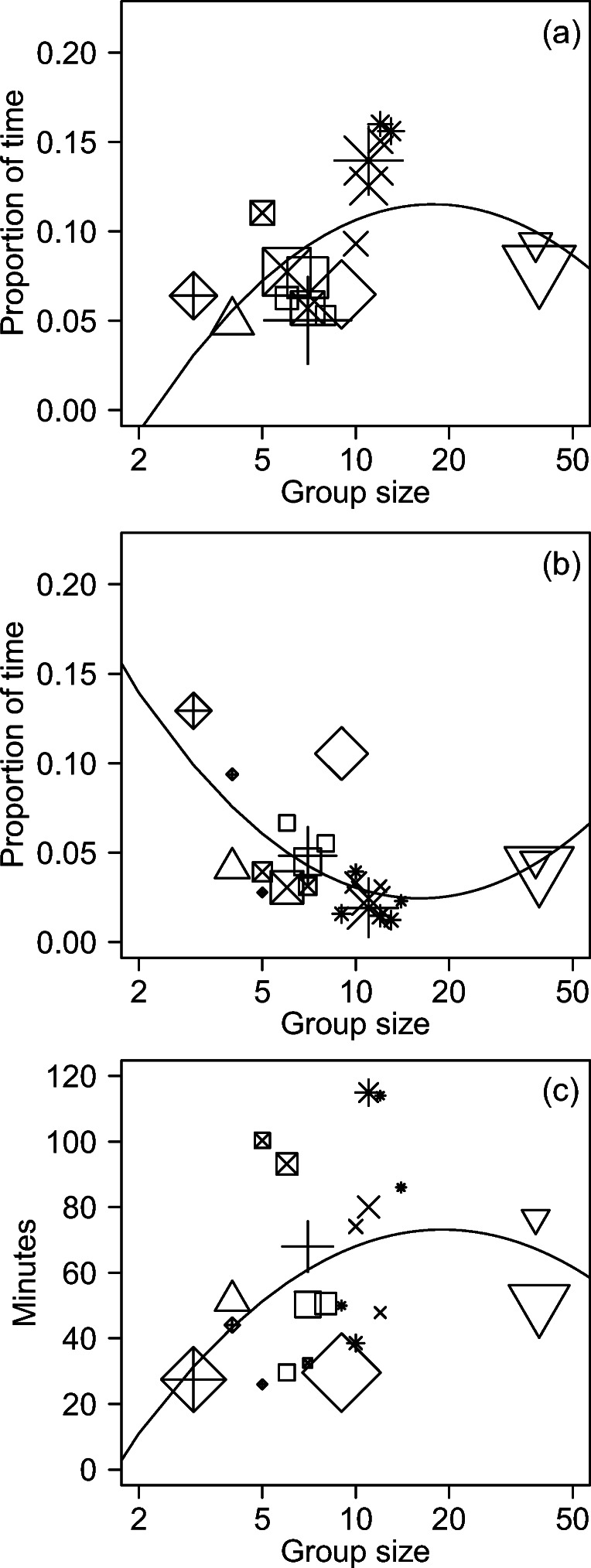
Proportion of time spent on asynchronous individual travel (**a**), proportion of time spent on synchronous group travel (**b**), and group patch residence times (**c**) versus the number of weaned gorillas in the group. The x-axes have a log-scale, and the y-axes have a linear scale. Lines are based on linear regressions of each response variable versus group size and size squared. Symbols represent different groups as listed in Fig. 2. Each data point represents a different size of a different group. The sizes of data points reflect differences in sample sizes (Table 2)

The 41 adult females in this study devoted 7.2 ± 2.8%SD of their time to asynchronous travel, which represents 67% of their total travel time. The remaining 33% of their travel coincided with movements by the rest of their group. The time budgets for individual asynchronous travel had a significant inverted U-shaped relationship with group size, which resembled our previous results for overall travel times and distances (Grueter et al. 2018). Asynchronous travel increased with size for most groups, before decreasing for the largest group (Table 4, c). Visual inspection again suggested that the quadratic term might be excessively sensitive to the data from the largest group, but the term remained significant when we removed that group from post-hoc analyses (Fig. 3a).

The average distance for asynchronous individual travel between food sites was 4.5 ± 5.8 SD meters. The average distance was 4.3 ± 4.7 m in the largest group, which is not significantly different from 4.9 ± 6.8 m in the two intermediate-sized groups (Table 4, d).

### Synchronous group travel and patch utilization

Group patch residence times had a median value of 51 min with an interquartile range of 23–106 min. Group patch residence times showed a significant inverted U-shaped relationship with group size (Table 5, a). Group patch residence times initially increased with group size, but then decreased for the largest groups (Fig. 3c). Those group patch residence times include only the time spent feeding, but they resemble the pattern for asynchronous travel, which may indicate that gorillas spent more time traveling within patches when they spent more time feeding in those patches. The results were not significant if we excluded the largest group.

**Table 5:** Results from statistical models for group patch residence times (a), proportion of time spent on synchronous travel (b), and distances for synchronous group travel (c)

Fixed effect	Estimate	StdErr	t/z	*p*
a) Patch residence times				
Variable	Estimate	StdErr	z	*p*
Group size	− 1.05	0.48	− 2.17	0.030
Size squared	0.84	0.35	2.41	0.016
b) Proportion of synchronous travel				
Variable	Estimate	StdErr	*t*	*p*
Intercept	0.55	0.14	4.00	–
Offset term	0.15	0.02	9.32	–
Group size	− 0.19	0.10	− 1.86	0.052
Size squared	0.18	0.07	2.57	0.009
Rainfall	− 0.02	0.02	− 0.98	–
Autocorrelation	0.11	0.02	7.03	–
c) Group travel distances				
Variable	Estimate	StdErr	*t*	*p*
Intercept	2.88	0.06	47.64	–
Size category	0.02	0.09	0.19	0.845
Autocorrelation	0.16	0.05	3.41	–

The proportion of time for synchronous group travel showed a significant U-shaped relationship with group size, with less frequent travel at intermediate group sizes (Table 5, b, Fig. 3b). Those results mirrored the patterns for group patch residence times and asynchronous individual travel: when groups spent more time feeding and traveling within patches, they spent less time traveling between patches. The patterns for synchronous and asynchronous travel do not offset each other completely, however, because the combined results for overall travel are more similar to the pattern for asynchronous travel (Grueter et al. 2018).

The average distance for a single episode of synchronized group travel was 25.8 ± 35.7 SD meters. Those distances averaged 24.4 ± 21.8 m for the largest group, which is not significantly different from 27.5 ± 39.4 for the two intermediate groups (Table 5, c).

The energy intake rate within food sites, the total energy intake per food site, and the food site residence times all declined significantly before the gorillas resumed synchronous group travel (potentially leaving a patch). The distance traveled to reach each food site was not changing significantly (Table 6). Collectively, those results are consistent with our predictions that foraging efficiency would decline as a group depleted the food within a patch. Gorillas were gaining energy more slowly, while obtaining less energy per food site and traveling the same distance between sites, so they would have needed greater effort to obtain the same amount of energy.

**Table 6:** Results from statistical models of foraging efficiency versus the time remaining until an episode of synchronous group travel (i.e., when the group was potentially leaving a patch). Energy intake rate, energy intake per food site, food site residence time, and distance traveled between food sites

Variable	Estimate	StdErr	*t*	*p*
a) Energy intake rate				
Intercept	2.705	0.097	27.891	
Offset	0.185	0.008	22.490	
Time	0.052	0.018	2.818	0.005
Autocorrelation	0.166	0.021	7.830	
b) Energy intake per food site				
Intercept	2.934	0.116	25.262	
Time	0.124	0.022	5.740	0.000
Autocorrelation	0.109	0.025	4.300	
c) Food site residence time				
Intercept	0.008	0.085	0.089	
Time	0.165	0.021	7.897	0.000
Autocorrelation	0.082	0.024	3.364	
d) Distance traveled between food sites				
Intercept	1.198	0.110	10.871	
Time	− 0.012	0.028	− 0.408	0.672
Autocorrelation	0.126	0.033	3.773	

The total energy intake per food site and the food site residence times both had a significant positive correlation with the elapsed time since a group stopped traveling synchronously (potentially entering a new patch). The elapsed time had a significant negative correlation with the distance traveled between food sites (Table 7). Collectively, those results would indicate that foraging efficiency increased as a group continued feeding in a patch, because gorillas were obtaining more energy from each food site while spending less time traveling from one food site to the next. Thus, the results are in the opposite direction of our predictions that foraging efficiency would decline as a group depleted the food within a patch. The energy intake rate within food sites was not significantly correlated with the elapsed time since a group stopped traveling synchronously.

**Table 7:** Results from statistical models of foraging efficiency versus the time since an episode of group travel (i.e., when the group was potentially entering a new patch). Energy intake rate, energy intake per food site, food site residence time, and distance traveled between food sites

Variable	Estimate	StdErr	*t*	*p*
a) Energy intake rate				
Intercept	2.814	0.094	30.031	
Offset	0.231	0.008	28.683	
Time	0.011	0.017	0.660	0.512
Autocorrelation	0.081	0.018	4.436	
b) Energy intake per food site				
Intercept	3.174	0.107	29.737	
Time	0.063	0.021	2.978	0.003
Autocorrelation	0.028	0.023	1.237	
c) Food site residence time				
Intercept	0.127	0.088	1.443	
Time	0.109	0.021	5.203	0.000
Autocorrelation	0.043	0.023	1.903	
d) Distance traveled between food sites				
Intercept	1.357	0.090	15.036	
Time	− 0.058	0.024	− 2.381	0.017
Autocorrelation	0.132	0.028	4.685	

## Discussion

### Group spread and asynchronous individual travel

The estimated “total occupied group area” while feeding was positively correlated with their number of weaned individuals (group size). Those results are consistent with expectations that large groups will have greater group spread, rather than compressing all of their individuals into the same area as small groups (Fig. 2a). Greater spread has been reported for larger groups of species such as gray-cheeked mangabeys (*Lophocebus albigena*), red colobus monkey (*Procolobus badius*), and ring-tailed coatis (*Nasua nasua*) (Gillespie and Chapman 2001; Chancellor and Isbell 2009a; Hirsch 2011; Gogarten et al. 2014).

The estimated “experienced density” of the focal gorilla within groups was positively correlated with the number of weaned individuals in the group, and the pattern was qualitatively similar to predictions from Watts (1991): the gorilla density rose gradually across most group sizes before increasing more dramatically with the largest group (Fig. 2b). Higher animal densities have also been reported within larger groups of teal (*Anas crecca*) and ring-tailed coati (*Nasua nasua*) (Poysa 1994; Di Blanco and Hirsch 2006). In contrast, the largest group of gray-cheeked mangabeys (*Lophocebus albigena*) had a lower density than the other groups, along with lower rates of agonism (Chancellor and Isbell 2009a). Animal density did not differ significantly between two groups of colobus monkeys, despite a two-fold difference in group size (Saj and Sicotte 2007). Thus, the effects of group size upon animal density (inter-individual spacing, number of neighbors, etc.) seem to show more variation than the effects upon group spread. The relationship between animal density and group spread can depend on how those terms are defined, as well as the geometric configuration of the group while feeding (Altmann 1974; Hirsch 2007).

Group size showed a significant inverted U-shaped relationship with the proportion of time spent on asynchronous individual travel (i.e., while the rest of the group was not traveling). Asynchronous travel increased with size for most of the groups, but then decreased with the largest group (Fig. 3a). Those patterns are consistent with our companion study of daily travel distances and the proportion of time spent on all travel (combining asynchronous individual travel and synchronous group travel) in this population (Grueter et al. 2018). Those previous results were attributed to increasing costs of within-group scramble competition for most groups, which was partially offset by benefits of between-group contest competition for the largest group (Grueter et al. 2018). If so, then our results suggest that most groups do not increase their group spread sufficiently to fully offset the first mechanism of scramble competition. Such results are consistent with predictions that the optimal group spread can involve a trade-off between feeding competition versus external threats including the risks of predation and/or infanticide by males outside the group (Watts 1991; Cowlishaw 1999; Smith et al. 2005; Di Blanco and Hirsch 2006).

### Synchronous group travel and patch utilization

The proportion of time for synchronous group travel decreased with group size among most groups, before increasing slightly for the largest group (Fig. 3b). For most groups, those results do not support our expectation that increases in group size would lead to greater increases in synchronous group travel than asynchronous individual travel. Nonetheless, the overall pattern for synchronous group travel is similar to the daily travel distance of woolly monkeys (*Lagothrix lagothricha*) and savanna baboons (*Papio cynocephalus*) (Stevenson and Castellanos 2000; Markham et al. 2015). In those species, the nonlinear pattern was considered evidence that larger groups must travel farther to overcome feeding competition within groups, but those costs are mitigated by their advantage in contest competition against smaller groups (Markham et al. 2015). Savannah baboons and mountain gorillas are both sexually dimorphic, so the competitive ability of their groups may be primarily determined by the quantity and quality of adult males, which could then be correlated with their number of adult females and total group members (Breuer et al. 2012; Markham et al. 2012; Grueter et al. 2018; Mirville et al. 2020).

Our estimates of group patch residence times were based on the hypothesis that gorillas were leaving a patch when they began synchronous group travel, and that they had reached a new patch when they stop traveling and resume feeding. If so, then our results would indicate that group patch residence times increased with group size for most groups, before decreasing for the largest group (Fig. 3c). Those results mirrored the patterns for synchronous group travel: when groups stayed longer in patches, they spent a lower proportion of time traveling between patches. Nonetheless, the results for most groups are in the opposite direction of expectations for the second mechanism for scramble competition, which predicts that larger groups will deplete patches more quickly (Janson and van Schaik 1988; Gillespie and Chapman 2001). Further study is needed to determine whether group patch residence times are also unexpectedly short for smaller groups of woolly monkeys and savannah baboons, whose overall travel times resembled our patterns for synchronous group travel (above). Theoretically, short group patch residence times could arise from intergroup competition if smaller groups are displaced from patches or relegated to lower quality habitats (Wrangham 1980; Janson and Goldsmith 1995; Majolo et al. 2008; Markham et al. 2013). If lower quality habitats have less food per patch, it could arise from smaller patches and/or lower food density within those patches.

Our analyses of foraging efficiency were limited to larger groups whose synchronous travel is less likely to be caused by intergroup competition. Based on our hypothesis that synchronous group travel occurs between patches, our results would indicate that foraging efficiency initially increases in a patch, but then it decreases before the group leaves. Although such a pattern is not entirely inconsistent with patch depletion, foraging efficiency is typically expected to decline monotonically as food becomes scarcer in a patch (Charnov 1976; Ginnett et al. 1999; Searle et al. 2005b). Instead, the results may reflect preexisting spatial variations in the quality of food that gorillas encounter as they traverse their habitat. If the food quality drops below a threshold level, groups may stop feeding and travel synchronously until it increases sufficiently again. Hypothetically, such gradual variations could involve patches of food that are surrounded by lower quality vegetation, but the distribution can also be the other way around (areas of lower quality vegetation that are surrounded by broader areas of food). Even slight declines in foraging efficiency might be sufficient to justify the short distances of synchronous group travel.

### Summary and conclusions

Our efforts to dissect the two mechanisms of within-group scramble competition became complicated by evidence of competition among groups, which has recently begun receiving greater consideration in studies of mountain gorillas (Seiler et al. 2017; Grueter et al. 2018; Mirville et al. 2020). Nonetheless, we were able to show that large groups typically had more asynchronous individual travel than small groups, even though they increased group spread to mitigate the first mechanism of scramble competition. Synchronous group travel seemed to reflect variations in foraging efficiency, but we found only limited evidence that patch depletion is causing the second mechanism of scramble competition. Collectively, the results did not support our expectation that increases in group size would lead to greater increases in synchronous group travel than asynchronous individual travel. The separate mechanisms of scramble competition may be more straightforward in studies without intergroup competition, especially if our temporal perspective on patches can be supplemented by direct observations of their physical boundaries (Altmann 1974; Chapman 1988; Janson and Goldsmith 1995; Searle et al. 2005a; Johnson et al. 2017).

Although this study was mainly intended to distinguish between the two types of scramble competition within the same species, portions of the results may pertain to species where only one type is common. For example, our results for asynchronous individual travel are probably most relevant for species where synchronous group travel is rare. Such species are expected to mitigate the first mechanism of scramble competition by increasing group spread, as observed in this study (Gillespie and Chapman 2001; Hirsch 2007; Saj and Sicotte 2007; Snaith and Chapman 2008; Chancellor and Isbell 2009b).

Studies of overall travel distances have suggested that scramble competition has less impact on folivores than frugivores, which is consistent with expectations that folivores may have greater potential to increase group spread, if their food is more evenly distributed than frugivores (Janson and Goldsmith 1995). Those generalizations have come under increased scrutiny, however, as evidence emerges that foliage is not always as evenly distributed as previously believed (Koenig et al. 1998; Saj et al. 2007; Snaith and Chapman 2007; Grueter et al. 2009). Thus, in addition to categorizing species according to their diet, it may be insightful to consider the proportion of their travel that is synchronous versus asynchronous. If groups can mitigate the costs of asynchronous individual travel by increasing group spread, then does scramble competition have greater impact on species whose travel is primarily synchronous?

## Data Availability

The datasets that were analyzed for this study are available in the Supplementary Information. The data is formatted as an .RData file for the “R” software, which is freely available online.

## Code availability

The computer code that was used to analyze the data is available from the corresponding author upon request.
